# Artificial Intelligence Models for the Detection of Microsatellite Instability from Whole-Slide Imaging of Colorectal Cancer

**DOI:** 10.3390/diagnostics14151605

**Published:** 2024-07-25

**Authors:** Gavino Faa, Ferdinando Coghe, Andrea Pretta, Massimo Castagnola, Peter Van Eyken, Luca Saba, Mario Scartozzi, Matteo Fraschini

**Affiliations:** 1 Dipartimento di Scienze Mediche e Sanità Pubblica, University of Cagliari, 09123 Cagliari, Italy; gavino.faa@unica.it; 2 UOC Laboratorio Analisi, AOU of Cagliari, 09123 Cagliari, Italy; fcoghe@aoucagliari.it; 3 Medical Oncology Unit, University Hospital and University of Cagliari, 09042 Cagliari, Italy; andrea.pretta@unica.it (A.P.); mario.scartozzi@unica.it (M.S.); 4 Laboratorio di Proteomica, Centro Europeo di Ricerca sul Cervello, IRCCS Fondazione Santa Lucia, 00179 Rome, Italy; maxcastagnola@outlook.it; 5 Division of Pathology, Genk Regional Hospital, 3600 Genk, Belgium; peter.vaneyken@kuleuven.be; 6 Department of Radiology, Azienda Ospedaliero Universitaria, University of Cagliari, 40138 Cagliari, Italy; luca.saba@unica.it; 7 Dipartimento di Ingegneria Elettrica ed Elettronica, University of Cagliari, 09123 Cagliari, Italy

**Keywords:** whole-slide images, microsatellite instability, colorectal cancer, artificial intelligence, deep learning

## Abstract

With the advent of whole-slide imaging (WSI), a technology that can digitally scan whole slides in high resolution, pathology is undergoing a digital revolution. Detecting microsatellite instability (MSI) in colorectal cancer is crucial for proper treatment, as it identifies patients responsible for immunotherapy. Even though universal testing for MSI is recommended, particularly in patients affected by colorectal cancer (CRC), many patients remain untested, and they reside mainly in low-income countries. A critical need exists for accessible, low-cost tools to perform MSI pre-screening. Here, the potential predictive role of the most relevant artificial intelligence-driven models in predicting microsatellite instability directly from histology alone is discussed, focusing on CRC. The role of deep learning (DL) models in identifying the MSI status is here analyzed in the most relevant studies reporting the development of algorithms trained to this end. The most important performance and the most relevant deficiencies are discussed for every AI method. The models proposed for algorithm sharing among multiple research and clinical centers, including federal learning (FL) and swarm learning (SL), are reported. According to all the studies reported here, AI models are valuable tools for predicting MSI status on WSI alone in CRC. The use of digitized H&E-stained sections and a trained algorithm allow the extraction of relevant molecular information, such as MSI status, in a short time and at a low cost. The possible advantages related to introducing DL methods in routine surgical pathology are underlined here, and the acceleration of the digital transformation of pathology departments and services is recommended.

## 1. Introduction

The introduction of whole-slide imaging (WSI), a technology that can digitally scan whole slides in high resolution, has significantly improved the efficiency of modern clinical pathology departments by facilitating all the steps related to tissue glass slide procedures. Moreover, the application of deep learning methods improved the productivity, accuracy, and reproducibility of pathological diagnoses [1,2,3].

Microsatellite instability (MSI) is a specific and uncommon genomic feature caused by a deficient mismatch repair (dMMR) system, occurring in approximately 12–15% of colorectal cancer (CRC), with a prevalence in females and older patients [4]. It is recommended that all patients presenting with CRC undergo MSI testing, given that MSI-CRCs are characterized by a high tumor mutational burden and by the overexpression of highly immunogenic neoantigens and immune checkpoints. Consequently, MSI allows the stratification of CRC patients, which has significant consequences on the therapeutic strategy that needs to be adopted [5]. In short, patients with MSI-CRC represent a distinct subset in whom standard treatments with fluorouracil-based chemotherapy are contraindicated. In contrast, therapy with immune checkpoint inhibitors and PD-1 blockade shows high efficacy [6,7].

MSI testing is also essential for identifying patients affected by Lynch syndrome, which is the hereditary cancer syndrome associated with germline mutations in MLH1, MSH2 and MSH6 genes [8]. Lynch syndrome is diagnosed in about 30% of carriers of CRC with MSI [9]. In clinical practice, testing for MSI is mainly based on immunohistochemistry (IHC) analyses for loss of MLH1, MSH2, MSH6, and PMS2. The absence of nuclear reactivity of cancer cells for all these proteins, or one of them, indicates a deficiency of the mismatch repair (dMMR) system and identifies the status of microsatellite instability (MSI). In cases with uncertain results at IHC, PCR and molecular tests are requested.

Unfortunately, these immunohistochemical and additional molecular methods may be very time-consuming and cost-intensive, leading to difficulties in their execution in clinical workflows, particularly in peripheral small hospitals and low-income and middle-income countries [10]. As a consequence, a large number of patients with CRC do not undergo screening for MSI, escaping relevant therapy strategies able to halt CRC progression despite guidelines recommending universal testing for MSI [8,11].

**Figure 1:**
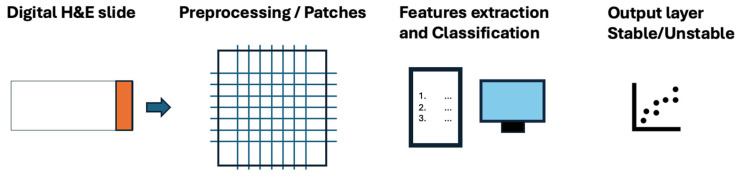
A schematic representation of the application of a generic AI model to MSI detection.

Deep learning (DL) models can detect MSI and dMMR in tumor samples on routine histology slides alone, representing a faster and less expensive tool than immunohistochemical and molecular assays. Embedding artificial intelligence (AI) models into diagnostic workflows might improve the screening of patients with a diagnosis of CRC, thereby reducing costly immunohistochemical and genetic tests and significantly increasing the speed at which data regarding MSI status are available to clinicians [12]. In Figure 1, we show a schematic representation of the application of a generic AI model to MSI detection.

Despite the high performance of AI-driven models when applied to histopathology, the digital transformation of pathology services is currently going very slowly, and many obstacles characterize the introduction of AI models into routine histopathology. Here, we report the most recent algorithms developed to extract relevant information from routine histological images, focusing on the detection of the MSI status from H&E-stained sections alone. We will also discuss the significant mental and practical obstacles that should be overcome to eventually allow the digital transformation of pathology departments and pathologists.

## 2. The Ongoing Road to Digitalization of Histopathology

### 2.1. Pathologists’ Role in Identifying Histological Predicts of Microsatellite Instability in CRC

A relevant step in the development of different approaches to the diagnosis of MSI based on the examination of H&E-stained sections has been played by histopathologic studies suggesting that microsatellite unstable colorectal cancer might show a distinctive histopathologic phenotype as compared with mismatch repair stable tumors. First, well-differentiated and focally mucinous tumors without necrotic cells in the lumen of tumor glands (so-called dirty necrosis) were reported to correlate with MSI [13]. Further studies suggested that the association of budding margins with a predominance of the mucous component in cancer cells might better predict microsatellite instability from CRC histology [14]. In a more recent study, the evaluation of ten histological changes reported to be associated with MSI–colorectal cancer in previous studies [15,16,17,18,19,20,21] revealed that only three histological features should be considered as predictive of the MSI status: (i) more than two tumor-infiltrating lymphocytes (TILs) per high-power field; (ii) the presence of mucinous differentiation in cancer cells; and (iii) the absence of dirty necrosis in the lumen of tumor glands.

Among these histological changes, mucinous differentiation was eventually reported to represent the strongest histological predictor of ground-truth MSI [22]. Despite these attempts to identify a phenotype of MSI colorectal cancer, based on the histology, the ability of gastrointestinal pathologists to identify the MSI status with appreciable reproducibility and specificity has remained low. In a recent study, the performance of five experienced gastrointestinal pathologists in detecting MSI on morphology, evaluated using the mean area under the receiver operating characteristic curve (AUROC) and the negative predictive value (NPV), was low. Moreover, the inter-reader agreement among pathologists in predicting the microsatellite instability status was low as well [22].

### 2.2. The Role of Deep Learning Models for the Identification of the MSI Status

Considering all these data taken together, a critical need emerged for developing a new broadly accessible and cost-efficient tool for testing MSI status. To this end, multiple artificial intelligence approaches, in particular deep learning models, have been proposed during the last five years to predict MSI directly from histology, looking for the identification of the optimal machine learning or deep-learning method for the prediction of response to immunotherapy in patients affected by CRC [23]. In a systematic review published in 2021, 58 full-text articles dealing with convolutional neural network (CNN)-based gastrointestinal cancer analyses, published between 2015 and 2020, were assessed for eligibility [24]. In a more recent systematic review in which the role of AI in predicting MSI status in CRC was evaluated, 17 articles published in PubMed up to June 2023 were included [25]. In this review, deep learning models for the MSI status showed a good performance in training cohorts with a mean AUC = 0.89. Here, the most relevant studies using deep learning models to identify the MSI status based on whole-slide images (WSIs) are reported.

Table 1 reports all the methods discussed in this work to identify the MSI status.

In 2019, Kather and coworkers were the first to provide evidence that a deep learning model might predict the MSI status utilizing H&E-stained sections in gastrointestinal cancer [26]. In this study, the algorithm Resnet18 was trained to classify MSI versus microsatellite stability (MSS) status in a large patient cohort from The Cancer Genome Atlas (TCGA), including 315 paraffin-embedded samples and 378 snap-frozen samples of CRC. The algorithm could distinguish features predictive of MSI status in paraffin-embedded as well as in frozen sections, allowing its use even on biopsies obtained during surgery. Interestingly, the model trained on paraffin sections showed the best performance on paraffin sections, whereas the model trained on frozen sections performed better when used on frozen samples. These findings first indicated the significant role of technical problems related to tissue samples’ treatment in performing the AI-driven models applied to histology.

MSINet is a deep learning model proposed by Yamashita and coworkers for predicting MSI in H&E-stained sections of CRC [22]. When applied to whole-slide images scanned at 20× and 40× magnification, this model exceeded the performance of experienced gastrointestinal pathologists at predicting MSI status. In this study, during the evaluation of the ten morphological features reported to be associated with MSI in colorectal cancer, only three were shown to be predictors of microsatellite instability: (i) more than two tumor-infiltrating lymphocytes per high-power field; (ii) mucinous differentiation; and (iii) the absence of necrotic cells in the lumen of cancer glands. Among these features, mucinous differentiation was the strongest predictor. Taking these data together, this model was indicated by the authors for an automated screening tool for the triage of all patients affected by CRC with a potential reduction in the number of classical immunohistochemical and molecular tests utilized in clinical practice for MSI, ending with substantial cost saving for the health system.

Echle and coworkers developed a deep-learning system that detects CRC specimens with dMMR or MSI using H&E-stained slides alone. Their algorithm detected the MSI status from digitized routine H&E-whole slide images with an AUROC of 0.96 in a large international validation cohort of 8836 colorectal tumors [27]. Interestingly, the deep learning system proposed by Echle et al. was validated in an external test set of patients intended to be population-based to mirror the clinicopathological characteristics of the general population. A finding of this study that appeared surprising was the ability to color-normalize the H&E-stained slides to improve the performance of the deep learning system. By applying the method previously developed by Macenko and coworkers [35], it was shown that color normalization can further improve both the performance and generalizability of the deep learning inference of MSI status in H&E-stained sections. Some limitations emerge from Echle’s study. The first regards the use of bioptic material: the performance of their algorithm was lower in bioptic samples due to the smaller tissue area compared with that of surgical specimens. Another limitation in the performance of the deep learning model was the false prediction of MSI status in samples with large necrotic or immune-infiltrated areas. Moreover, a false negative prediction was associated with technical artifacts, resulting in a blurred image, suggesting that improved quality control of the scanned slides could increase the performance of the deep learning assay. In further articles on the application of AI models to MSI detection in CRC, Echle et al. focused on the performance of the algorithm, showing that AI systems can be applied as a rule-out test for MSI by using cohort-specific thresholds with a fixed sensitivity at 95% [28]. In this study, the authors tried to identify the reasons for misclassification, focusing on the 22 out of 8343 patients analyzed in whom the algorithm gave a false negative result. Among the 22 false negative cases, at a revision performed by two expert gastrointestinal pathologists, eight (36%) had no tumor or a very small portion of cancer; seven (32%) were characterized by technical artifacts, such as overstaining or folds; and in the remaining eight cases (36%), no reason was identified at histological revision. These findings stress the need for practical guidelines for the optimal quality of slides to be scanned for AI diagnostic testing.

A novel deep learning framework designed for predicting the status of key molecular pathways and mutations in CRC, involving three convolutional neural network models, was introduced by Bilal and coworkers in 2021 [29]. The training of this algorithm was not restricted to the MSI status, including the hypermutation status, chromosomal instability, BRAF, TP53 and KRAS mutations. Following a large-scale validation, the authors proposed their algorithm for predicting microsatellite instability, key molecular characteristics and the mutation status of key genes such as KRAS, BRAF and TP53 in CRC patients. The use of their convolutional neural network model, called HoVer-Net, allowed the authors to classify cancer samples into five categories: (i) neoplastic epithelial cells; (ii) non-neoplastic epithelial cells; (iii) inflammatory cells; (iv) mesenchymal cells; and (v) necrotic cells. Microsatellite instability was associated with a high proportion of tumor-infiltrating inflammatory cells and necrotic cancer cells and a relatively low proportion of neoplastic epithelial cells and mesenchymal cells. The high proportion of tumor-infiltrating lymphocytes (TILs) was indicated as the strongest association with MSI in colorectal cancer. On this basis, the authors suggested using their algorithm (HoVer-Net) in clinical practice to stratify CRC patients for targeted therapies. The lower costs of the deep learning approach and the quicker turnaround time, compared to immunohistochemical or sequencing-based traditional methods, were underlined by the authors in their paper.

A deep learning-based method called DeepSMILE (Deep Self-supervised Multiple Instance Learning) was developed for analyzing whole-slide images of H&E-stained tumor sections by Schirris and coworkers [30]. MSI prediction was performed with this algorithm with a 0.87 AUROC showing that the standard use of self-supervised learning techniques, combined with multiple instance learning, may improve genomic label classification performance in the histopathology domain.

An interesting article from Niehues and coworkers [31] compared six different deep learning architectures to predict multiple biomarkers, including MSI, from the histopathology of CRC. In this study, a large external validation cohort was utilized in order to provide a realistic evaluation setting. AI-based models utilizing self-supervised, attention-based and multiple-instance learning outperformed other approaches. Regarding the prediction of the MSI status and BRAF mutations, the best deep learning method achieved clinical-grade performance. On the contrary, the mutation prediction of KRAS, NRAS and TYP53 was insufficient for clinical purposes.

The deep learning model proposed by Jiang and coworkers [32] is based on multiple instance learning (MIL) techniques and shows a sensitivity of 90% and a specificity of 95% in predicting the mismatch repair (MMR) status in CRC based on H&E-stained sections alone. This MIL model showed high sensitivity and specificity in surgical specimens confirmed even in bioptic samples. Finally, in this study, a dual-threshold triage strategy was proposed to minimize the number of immunohistochemical tests for MSI.

MSIntuit is an AI-based algorithm developed as a screening tool for MSI detection from CRC histology slides [33]. This algorithm was specifically developed for working in clinical workflows to ease dMMR/MSI testing and accelerate oncologists’ decision making in routine clinical practice. It reached a sensitivity of 0.98 and a specificity of 0.47. An interesting finding emerging from this study was the analysis of the inter-scanner variability by digitizing each histological slide using two different scanners. MSIntuit yielded an excellent inter-scanner agreement, and according to the authors, it could effectively serve as a pre-screening tool that could alleviate the MSI testing burden.

Gerwert and coworkers have proposed an innovative digital pathology approach to diagnosing MSI status [34]. This model was based on artificial intelligence-integrated infrared imaging, utilizing unstained paraffin sections observed using a quantum cascade laser (QCL) microscope.

### 2.3. Algorithm Sharing: New Approaches to Decentralize Artificial Intelligence in Histopathology

One of the principal concepts for people approaching the application of AI-driven models for diagnostic purposes is the assumption that “performance of deep learning models significantly increases training thousands rather than hundreds of data”. This means that classifier performance increases significantly when the sample size increases by approximately 10,000 patients [10,12,36]. Consequently, training AI models devoted to an application for clinical purposes usually requires the data sharing of patient-related features, including whole-slide images of histological samples, with other institutions across different countries. In clinical practice, such data sharing faces multiple logistical and legal obstacles, including those related to privacy and ethical committees, that may halt the development of deep learning models with high sensitivity and specificity, which are qualities requested for clinical applications.

The first answer to these problems came from centralized federal learning (FL) [37,38]. According to this new approach, in an FL project, multiple deep learning models may be trained independently on separate computers in the different centers participating in the project, which do not share clinical data but only share the learned model weights. In FL, a central coordinator supervises and controls the learning process. FL enabled the privacy preservation and cooperation of multiple partners on the training of AI models. However, the presence of a central coordinator center with information governance resulted in the concentration of the power of exploitation in the hands of a single center, halting the process of strict cooperation necessary to reach a high number of patients to increase the performance of each AI model.

The swarm learning (SL) approach is characterized by the absence of central control and coordination so that the decentralization and protection of confidential clinical data are assured [39]. In SL, models are trained locally and combined centrally, raising all participants to the same level, removing the centralization typical of the FL approach and avoiding the concentration of data in one center. SL aims to facilitate collaboration among multiple clinical centers and generate more robust and reliable AI models, improving their quality and robustness, which is an indispensable prerequisite for their introduction in clinical practice. SL may offer a solution to the governance acquired with a deep learning model, including whole-slide images from histopathology. Moreover, SL facilitates a truly collaborative approach, in which partners from different clinical centers, including peripheral hospitals and pathology services from low-income countries, may communicate and work at the same level, cooperating to train AI models and share the benefits. The final goal is to introduce AI methods into routine diagnostic workflows worldwide [10].

### 2.4. Bias and Limitations of Deep Learning Approach to Histopathology

Deep learning-based analyses of histological images show many advantages, including high prediction abilities and the extraction of routine histology of molecular data not available at microscopy. On the other hand, many biases and limitations have been evidenced in the literature over the years. The introduction of AI-driven models in the clinical workflow needs an explanation of these biases and limitations to resolve common challenges encountered in DL-digital pathology and increase the performance of the models. Crucial bias, which may limit the application of deep learning models in clinical histopathology, may be hard to detect. They may be subdivided into three subgroups: (1) dataset bias, (2) bias correlated with class labels, and (3) sampling bias.

Dataset bias affects the entire dataset spread across all classes, including test data, and is often introduced when creating datasets. This bias may be uncovered using a dataset where labels exclusively depend on the cell(s) in the patch’s center independently of any other cell or feature occurring in the patch.

Bias correlated with class labels, the second type of bias, is related to image features, which are unintentionally correlated with the labels of the dataset. This bias may occur from artifacts or cell types that often co-occur with the label.

Sampling bias, the third type of bias, is mainly related to the training data, which should be representative of the tissue analyzed and contain all possible sample classes. Since neural networks are trained on only a few regions of interest (ROIs) of the whole-slide image, the model should be trained on all representative parts of the whole image so that the model may be applied on WSIs at test time [40].

Some limitations of AI-driven models have been associated with the patch-based learning method utilized to capture cell details, which are not detected in WSIs [41]. These biases have been, at least in part, solved by the introduction of methods for training neural networks on entire WSIs [42].

The absence of validation of algorithms and computational technologies represents a significant obstacle to the applicability of AI models in clinical workflows. Marked variations regarding slide preparation, scanner models and digitalization from one center to another are the basis of differences in the performance of the different single-center methods [43].

The absence of algorithm elucidation is generally considered a barrier to the acceptance of AI-driven models by the medical community [44]. To this end, new approaches are requested to elucidate how DL-based algorithms operate to obtain pathologists’ and clinicians’ trust.

The file size of histological images is about 1000 times higher than that of X-ray images. Consequently, hardware with robust computing infrastructure and large storage capacity is requested. The development of the global widespread 5G network may represent the solution to this important problem [43].

Pathologists and clinicians, particularly oncologists, should be trained to utilize deep learning models and the new approach to histopathology. Moreover, their willingness to accept results performed by “machines” should be encouraged toward a collaboration between machines and humans to be applied in clinical practice. In this project, clinical laboratory doctors should be indicated as a model. A shift from the procedure of the exclusive use of the microscope toward a diagnosis performed at the computer screen on digitized histological images appears mandatory. Regarding pathologists worried about signing a diagnosis performed by a machine (something routinary in clinical labs), molecular tests extracted by H&E-stained sections should be verified, in a period of passage, through the classical immunohistochemical or molecular tests in order to encourage their introduction for clinical purposes.

Large training cohorts will likely boost the classification performance of the various deep learning models proposed in the literature. Moreover, in large cohorts of patients, rare morphological variants of each tumor type can be learned by the network, incrementing its diagnostic abilities [26]. Multi-center studies and the external validation of one center data should be encouraged. Deep learning models developed to outperform genetic or molecular tests on histological slides necessitate training on large cohorts of patients with clinical endpoints and genetic or molecular characterization with the tests validated in clinical settings [27].

The normalization of H&E-stained histological images before quantitative analyses is mandatory [35]. Native, non-normalized slides may show marked differences regarding staining hue and intensity. Color normalization has been shown to improve specificity and sensibility levels in the detection of microsatellite instability on H&E-stained sections in colorectal tumors [27]. Regarding the staining method utilized, most studies on WSIs are carried out on H&E-stained sections. AI-models’ performance might be improved by utilizing additional histological and immunohistochemical stains [23].

AI models, contrary to molecular and immunohistochemical methods, are often not robust enough to differing tissue or patient characteristics. This issue has been well evidenced by the different performances observed when a model trained in a center was applied to datasets from another center. This finding represents a major barrier to the widespread diffusion and implementation of deep learning models in human pathology [23].

## 3. Conclusions

At these times, pathologists have something very impressive going on in their discipline: artificial intelligence. In this review, we tried to channel to the readers all the most important studies that, in the last five years, have shown that deep learning-based models can reliably detect MSI status based on routine histology alone. The application of AI to routine diagnostic pathology might profoundly affect the diagnosis and therapy of multiple tumors, including CRC. According to the data reported here, a deep learning-based prediction of MSI status will provide added value to correct clinical testing paradigms. Deep learning methods are significantly faster and less expensive than routine laboratory assays. Therefore, the future integration of deep learning methods in the clinical workflow has the potential to reduce the molecular testing load and enable universal and low-cost MSI testing from routine material, such as H&E-stained sections.

The discrepancy between the possible key role of deep learning models in extracting multiple biomarkers useful for prognosis and therapy and the restriction of their application to high-level research centers indicates the many challenges to be addressed before the clinical implementation of deep learning-based pathology. On the other hand, deep learning concepts and the deep learning mentality will have tremendous benefits for diagnostic and therapeutic decisions in oncology in the near future.

Within this AI-driven revolution regarding the whole medical world, deep learning-assisted histopathology would play a key role, allowing pathologists to give relevant information on the mutational status of each tumor and H&E-stained histological sections converted into digital images with a slide scanner. So, relevant information might be given to oncologists in a very short time and without any significant addictive cost. Thanks to the internet network, every pathologist in every hospital might give his/her patient an expert histopathological and molecular diagnosis, utilizing a deep learning model developed for this task.

One central point that should be solved to reach this goal is the fear of pathologists, who should accept that DL models outperform expert pathologists in the classification of histological images and employ AI-based systems as powerful assistance tools in their routine clinical practice [24,45]. From a practical point of view, in order to give a reassuring answer to pathologists working on the possibility of inserting in the diagnostic protocol the diagnosis of “microsatellite instability” only based on a machine learning model, a confirmation test based on immunohistochemistry or molecular tests should be required for all cases that are positive in the AI test [46]. This means that the trained algorithm should be utilized by pathologists in triaging all CRC, excluding further negative test cases in the AI test, resulting in cost savings for health systems worldwide. In the future, it would be essential to consider expanding the available clinical datasets to improve the generalizability of the outcomes, optimizing tissue processing protocols to mitigate performance differences and, more generally, conducting a deeper analysis of misclassification causes to develop targeted performance enhancement strategies.

## Figures and Tables

**Table 1:** The list of all the methods discussed in this work.

Authors	Year	AI Model
Kather et al. [26]	2019	CNN with deep residual learning (resnet18)
Yamashita et al. [22]	2021	MSINet: a modified MobileNetV2 architecture
Echle et al. [27]	2020	A modified ShuffleNet deep learning system
Echle et al. [28]	2022	ResNet18 neural network model
Bilal et al. [29]	2021	Model 1 (ResNet18) and model 2 (adapted ResNet34)
Schirris et al. [30]	2022	Self-supervised pre-training and feature variability-aware deep multiple instance learning
Niehues et al. [31]	2023	Compared six different DL architectures to predict biomarkers from pathology slides
Jiang et al. [32]	2022	Densenet121 integrated with focal loss
Saillard et al. [33]	2023	A variant of the Chowder model
Gerwert et al. [34]	2022	CompSegNet and VGG 11 Neural Network

## Data Availability

Not applicable.
